# Observing the two-dimensional Bose glass in an optical quasicrystal

**DOI:** 10.1038/s41586-024-07875-2

**Published:** 2024-09-11

**Authors:** Jr-Chiun Yu, Shaurya Bhave, Lee Reeve, Bo Song, Ulrich Schneider

**Affiliations:** 1 Cavendish Laboratory, University of Cambridge, Cambridge, UK; 2 Material and Chemical Research Laboratories, Industrial Technology Research Institute, Hsinchu, Taiwan; 3 State Key Laboratory for Mesoscopic Physics and Frontiers Science Center for Nano-optoelectronics, School of Physics, Peking University, Beijing, China

**Keywords:** Ultracold gases, Phase transitions and critical phenomena

## Abstract

The presence of disorder substantially influences the behaviour of physical systems. It can give rise to slow or glassy dynamics, or to a complete suppression of transport as in Anderson insulators^1^, where normally extended wavefunctions such as light fields or electronic Bloch waves become exponentially localized. The combined effect of disorder and interactions is central to the richness of condensed-matter physics^2^. In bosonic systems, it can also lead to additional quantum states such as the Bose glass^3,4^—an insulating but compressible state without long-range phase coherence that emerges in disordered bosonic systems and is distinct from the well-known superfluid and Mott insulating ground states of interacting bosons. Here we report the experimental realization of the two-dimensional Bose glass using ultracold atoms in an eight-fold symmetric quasicrystalline optical lattice^5^. By probing the coherence properties of the system, we observe a Bose-glass-to-superfluid transition and map out the phase diagram in the weakly interacting regime. We furthermore demonstrate that it is not possible to adiabatically traverse the Bose glass on typical experimental timescales by examining the capability to restore coherence and discuss the connection to the expected non-ergodicity of the Bose glass. Our observations are in good agreement with recent quantum Monte Carlo predictions^6^ and pave the way for experimentally testing the connection between the Bose glass, many-body localization and glassy dynamics more generally^7,8^.

## Abstract (web summary)

The two-dimensional Bose glass state of matter is realized experimentally using ultracold atoms in an eight-fold symmetric quasicrystalline optical lattice, and the phase transition between Bose glass and superfluid is directly observed.

## Main

The interplay between disorder and interaction is central to the richness of condensed-matter physics as any real-life material will inevitably contain a certain degree of impurities and defects, and interparticle interactions are almost always present. While disorder tends to localize non-interacting particles, leading to Anderson localization^1^, interactions can counteract this, resulting in conducting ergodic states. More generally, the combination of disorder and interactions gives rise to rich physics governed by reduced or absent relaxation and transport, such as glassy dynamics or non-ergodic many-body localized systems, and forms one of the central topics in quantum statistical physics during the past decade^2^.

**Fig. 1:**
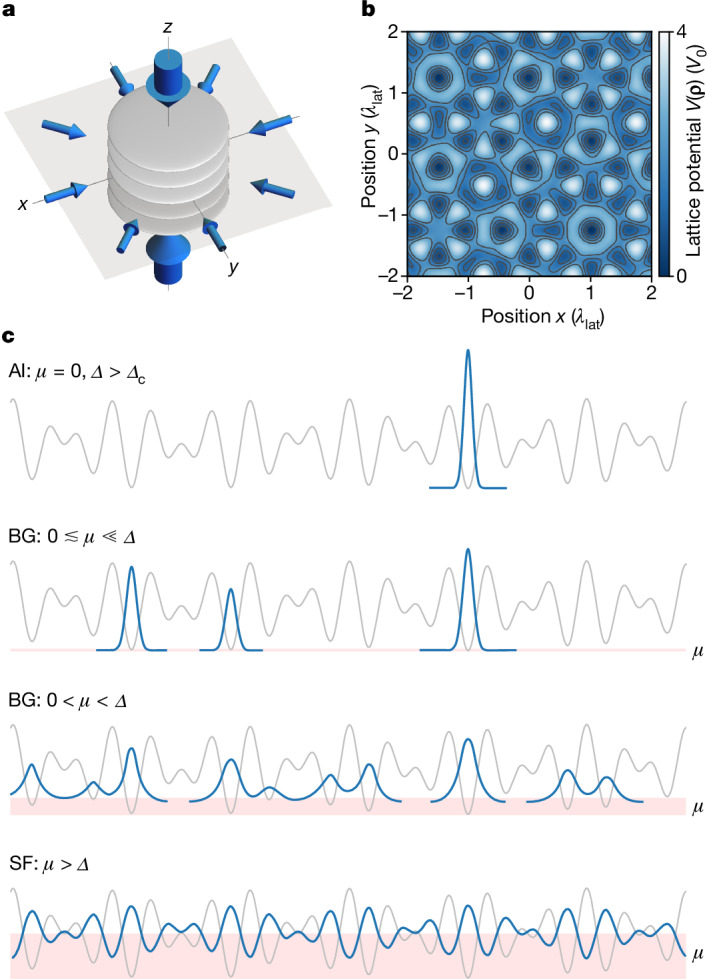
Lattice potential and sketch of possible phases. **a**, The 2D quasicrystalline optical lattice is generated by superimposing four independent 1D lattices in the *x*–*y* plane, marked by small arrows. A deep *z* lattice (large arrows) divides the system into a series of independent quasi-2D layers. **b**, Exemplary potential in a single layer. **c**, Repulsive interactions can delocalize an originally localized disordered system. From top to bottom, the sketches show the transition of the system’s ground state with increasing chemical potential *μ*, starting from the Anderson insulator (AI) in the non-interacting limit (*μ* = *ϵ*_0_ = 0), where the disorder strength *Δ* is above the critical disorder strength for localization *Δ*_c_, over the localized but compressible Bose glass (BG) for weak repulsive interactions where bosons spill over into other low-lying minima and form local superfluid puddles, into the superfluid (SF) when the chemical potential is comparable to or larger than the disorder strength *Δ*.

In bosonic systems, a hallmark of this interplay is the emergence of an additional ground-state phase called Bose glass. The Bose glass is an insulating but compressible phase without long-range phase coherence^3,4^. It was originally discussed purely as a ground state at zero temperature, but has been shown to extend to finite energy^9–12^. In the weakly interacting regime, the Bose glass can be understood by starting from a non-interacting Anderson insulator, where in the ground state all bosons localize at the lowest potential minimum (Fig. 1c). Adding small repulsive interactions to such systems will lead to bosons spilling over into other low-lying orbitals to minimize the interaction energy. This regime has also been referred to as an Anderson glass or Lifshitz glass^13,14^. With increasing interactions or density, and thereby increasing chemical potential, these originally isolated orbitals will form local superfluid puddles that will eventually merge into a global superfluid phase.

As the lowest-lying minima are typically located arbitrarily far away from each other, any changes or relaxation processes that require redistribution of particles between these distant minima may, in localized phases, require arbitrarily long times. In the non-interacting Anderson limit, orbitals localized at different local minima can indeed possess arbitrarily close energies while having only exponentially weak couplings^15^, thus resulting in many almost degenerate levels. This absence of level repulsion is a hallmark of non-ergodic phases^16,17^. As a consequence of these exponentially small gaps, even rather slow parameter changes within the Bose glass could be expected to take the system out of equilibrium.

In fact, although local changes within a localized system can induce changes over distances that are large compared with the localization length, the characteristic distance could be shown to increase only logarithmically with time and thus directly leads to exponentially large timescales in large systems^18^. Therefore, the thermodynamic notion of quasistatic or adiabatic changes, where the system remains in thermal equilibrium at all times and the process is isentropic, may not apply. This is reminiscent of many-body localization (MBL)^2^ and opens the question to which degree the Bose glass can be seen as the low-energy limit of a more general potential bosonic MBL phase.

Disordered interacting bosons have been studied for instance using helium-4 in porous media^19^, Cooper pairs in superconducting films^20^ and disordered quantum magnets^21^. In the context of ultracold atoms, the Bose glass has been extensively studied using various numerical methods^22–30^. Previous experiments in one dimension have shown the loss of coherence but were strongly affected by finite-temperature effects^31–35^ and experiments in three dimensions using speckle disorder studied momentum and quench responses^36,37^.

In this work, we investigate the low-energy states of a weakly interacting Bose gas in a two-dimensional (2D) eight-fold rotationally symmetric quasicrystalline optical lattice^5^. By analysing the momentum distribution of the system, we observe the Bose-glass-to-superfluid phase transition, and map out the phase diagram in the weakly interacting regime. Furthermore, our work experimentally establishes the non-adiabatic nature of the Bose glass, thereby highlighting its continuous connection to potential bosonic MBL phases at finite energy density^7,8^.

## A 2D quasicrystalline optical lattice

Quasicrystals are long-range ordered yet not periodic^38,39^ and thereby represent a fascinating middle ground between order and disorder. In contrast to purely random potentials, where in one and two dimensions all single-particle eigenstates are localized for any non-vanishing disorder^40^, quasiperiodic potentials support a phase transition from extended to exponentially localized states at a finite potential depth^41,42^, thus providing an ideal platform for studying disorder-induced phenomena.

In our experiment, we load a degenerate Bose gas of about 1.2 × 10^5^ potassium ^39^K atoms without discernible thermal fraction into a 2D quasicrystalline optical lattice using a 45-ms-long exponential ramp (Methods). The optical quasicrystal is formed by superimposing four independent blue-detuned one-dimensional (1D) lattices in the *x*–*y* plane at 45° angles, as depicted schematically in Fig. 1a. Each of these lattices is a 1D standing wave created by a retro-reflected laser beam at wavelength *λ*_lat_ = 725.4 nm. In addition, a deep lattice along the direction perpendicular to the plane (*z* axis) effectively slices the system into an array of 2D layers (see the grey disks in Fig. 1a). The resulting potential is given by

1 $$\begin{array}{ccc} &  & V({\boldsymbol{\rho }}=\{x,y\},z)={V}_{0}\mathop{\sum }\limits_{i=1}^{4}{\sin }^{2}({{\bf{k}}}_{i}\cdot {\boldsymbol{\rho }}+{\phi }_{i})+{V}_{z}{\sin }^{2}({k}_{z}z),\\  &  & {{\bf{k}}}_{i}\in \frac{2{\rm{\pi }}}{{\lambda }_{{\rm{l}}{\rm{a}}{\rm{t}}}}\left\{\left(\begin{array}{c}1\\ 0\end{array}\right),\frac{1}{\sqrt{2}}\left(\begin{array}{c}1\\ 1\end{array}\right),\frac{1}{\sqrt{2}}\left(\begin{array}{c}-1\\ 1\end{array}\right),\left(\begin{array}{c}0\\ 1\end{array}\right)\right\},\end{array}$$

where *V*_0_ and *V*_*z*_ denote the lattice depths, and **k**_*i*_ and *k*_*z*_ are the respective wavevectors ($|{{\bf{k}}}_{i}|={k}_{z}={k}_{{\rm{l}}{\rm{a}}{\rm{t}}}=2{\rm{\pi }}/{\lambda }_{{\rm{l}}{\rm{a}}{\rm{t}}}$) of the four 1D lattices in the *x*–*y* plane and the *z* lattice. The phase offsets *ϕ*_*i*_ are central to describe phasonic degrees of freedom and topological pumping in these potentials, but have no significant role in localization in large systems^43^.

**Fig. 2:**
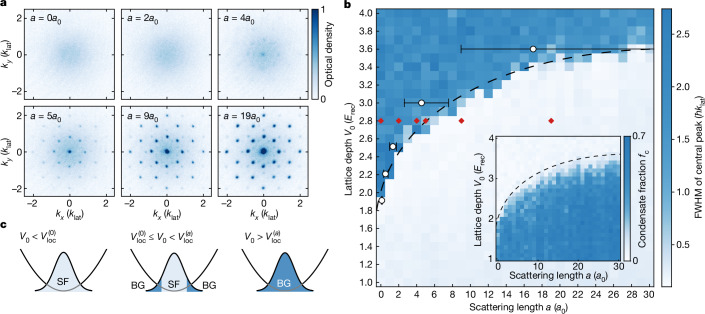
Bose-glass-to-superfluid transition. **a**, TOF images (9 ms TOF, 5 shots averaged) for different scattering lengths *a* at a fixed lattice depth of *V*_0_ = 2.8 *E*_rec_. Although the system is localized in the non-interacting and very weakly interacting cases, the appearance of sharp interference peaks for stronger interactions signals the emergence of long-range phase coherence, characteristic for the superfluid. **b**, Width of the central peak, distinguishing the coherent superfluid (light blue) from the incoherent Bose glass (dark blue). The dashed line is a guide to the eye indicating the detected phase boundary in the centre of the cloud ${V}_{{\rm{loc}}}^{(a)}$. It is identical to the line shown in the inset and in Fig. 3d. White points and error bars denote the QMC prediction from ref. ^6^ (Methods). Images in **a** correspond to the parameters marked by red diamonds. The inset shows the condensate fraction *f*_c_ extracted from the same set of images, highlighting the coexistence of the two phases. **c**, Phase transition in an inhomogeneous system. The shaded Gaussian denotes the in-trap atomic density and the parabola represents the external trapping potential. For shallow lattices, the ground state is purely superfluid (left). At the non-interacting critical depth ${V}_{{\rm{loc}}}^{(0)}$, the Bose glass starts to appear at the low-density edge of the cloud where interaction effects are small (middle). With increasing lattice depth, the phase boundary gradually moves inwards until the entire cloud enters the Bose glass phase at ${V}_{{\rm{loc}}}^{(a)}$ (right).

Throughout this work, the depths of the horizontal lattices are varied in the range of *V*_0_ = 1–4 *E*_rec_ and the *z* lattice is kept at *V*_*z*_ = 20 *E*_rec_, where ${E}_{{\rm{rec}}}={\hbar }^{2}{k}_{{\rm{lat}}}^{2}/(2m)$ is the recoil energy, *ħ* is the reduced Planck constant and *m* is the atomic mass. The deep *z* lattice provides a sufficiently strong vertical confinement so that interlayer tunnelling is negligible. As a consequence, atoms loaded into the lattice will be tightly confined to individual quasi-2D systems that show an eight-fold symmetric quasicrystalline structure, as depicted in Fig. 1b. A red-detuned dipole trap (Methods) provides an overall harmonic confinement and gives rise to an inhomogeneous density distribution (Fig. 2c).

Even though the lattice depths used for the 2D quasicrystalline lattice are rather low, the physics of the system is nonetheless captured by a dedicated quasiperiodic Bose–Hubbard (QBH) model^43^, which in second quantization reads

2 $${\widehat{H}}_{{\rm{QBH}}}=\sum _{i}{{\epsilon }}_{i}{\widehat{a}}_{i}^{\dagger }{\widehat{a}}_{i}-\sum _{i\ne j}{J}_{ij}{\widehat{a}}_{i}^{\dagger }{\widehat{a}}_{j}+\sum _{i}\frac{{U}_{i}}{2}{\widehat{n}}_{i}({\widehat{n}}_{i}-1).$$

Here ${\widehat{a}}_{i}^{\dagger }$ (${\widehat{a}}_{i}$) is the bosonic creation (annihilation) operator on the *i*th lattice site, and ${\widehat{n}}_{i}={\widehat{a}}_{i}^{\dagger }{\widehat{a}}_{i}$ is the corresponding number operator. The Hamiltonian ${\widehat{H}}_{{\rm{QBH}}}$ is characterized by three site-dependent parameters, namely, on-site energies *ϵ*_*i*_ (neglecting the harmonic confinement), tunnelling energies *J*_*ij*_ and on-site interactions *U*_*i*_ ∝ *a*, whose scale can be independently controlled by tuning the atomic s-wave scattering length *a* by means of a Feshbach resonance (Methods). We set ${{\epsilon }}_{0}:= \min \,{{\epsilon }}_{i}=0$ and use $\varDelta := \max \,{{\epsilon }}_{i}$ as an intuitive measure of ‘disorder strength’, even though the modulation in *J*_*ij*_ and *U*_*i*_ also influences the physics.

In the weakly interacting regime, systems described by the Hamiltonian ${\widehat{H}}_{{\rm{QBH}}}$ host a phase transition from Bose glass to superfluid, as illustrated in Fig. 1c. At strong interactions with *U* ≫ *J*, they furthermore host commensurate Mott insulators^6,43^; however, this regime is not probed in the current paper (Methods). In this strongly interacting regime, the term Bose glass was introduced to describe the phase emerging when the charge order of the Mott insulator vanishes for strong enough disorder *Δ* ≈ *U* (refs. ^25,29^). This regime shows the same phenomenology as the weakly interacting Bose glass, namely being a compressible, gapless, insulating phase without long-range coherence, and hence they both belong to the Bose glass phase.

## Phase diagram

Our main observable to distinguish superfluid and localized states is the momentum distribution detected using time-of-flight (TOF) imaging, that is, by releasing the atomic cloud from all trapping potentials and imaging its density distribution after 9 ms of free expansion. This can be understood as a matter-wave diffraction experiment where waves originating on different lattice sites expand, overlap and then interfere. Analogous to diffraction experiments in optics and in periodic lattices^44,45^, the coherence length, which quantifies the range of spatial coherence between lattice sites, determines the width of the matter-wave interference peaks. A high-contrast interference pattern composed of sharp peaks indicates the presence of long-range phase coherence, the signature of the superfluid phase. Localized states with only short-range coherence, however, result in an incoherent broad momentum distribution.

Figure 2a shows a series of TOF images recorded for different scattering lengths at a fixed lattice depth of *V*_0_ = 2.8 *E*_rec_. At this lattice depth, the single-particle ground state is strongly localized^46^, and the measured momentum distribution at vanishing scattering length (top-left panel) correspondingly shows the broad momentum profile of a localized Anderson insulator. With increasing interactions, however, we observe the emergence of initially faint but sharp interference peaks, signalling the phase transition from the incoherent Bose glass to a superfluid in the high-density core of the cloud. The remaining broad background corresponds to the incoherent Bose glass at lower densities, where the critical lattice depth is lower and approaches the non-interacting limit.

To quantitatively study this transition at the high-density centre of the cloud, we choose an observable that can detect the presence of even a small superfluid component, namely, the full-width at half-maximum (FWHM) of the central peak. The FWHM, extracted from 2D Gaussian fits, provides an almost binary signature: if there exists a superfluid component, the FWHM corresponds to the width of the superfluid peak, which is dominated by the in situ cloud size^47^ (Methods). Only when the superfluid completely vanishes, the FWHM jumps to the width of the incoherent background (Methods and Extended Data Fig. 3).

The resulting phase diagram for the centre of the trap is shown in Fig. 2b and clearly reveals two distinct phases: the coherent superfluid at shallow lattices (light blue) turns relatively abruptly into the incoherent Bose glass (dark blue) at an interaction-dependent critical lattice depth ${V}_{{\rm{loc}}}^{(a)}$. At vanishing scattering length, the observed ${V}_{{\rm{loc}}}^{(0)}$ coincides with the known single-particle localization point at around ${V}_{{\rm{loc}}}^{(0)}=1.78(2)\,{E}_{{\rm{rec}}}$ (refs. ^41,46^) up to minor corrections (≲1*a*_0_, where *a*_0_ denotes Bohr's radius) stemming from the presence of weak residual interactions due to small dipole–dipole interactions^48^ and calibration uncertainties (Methods). With increasing scattering lengths, the critical lattice depth ${V}_{{\rm{loc}}}^{(a)}$ indicated by the dashed line shifts considerably towards deeper lattices, directly demonstrating that even weak repulsive interactions can significantly counteract localization. The observed transition agrees well with the recent quantum Monte Carlo (QMC) simulations for the ground state reported in ref. ^6^. These low localization thresholds also imply large tunnelling energies^43^ and hence a high resilience to temperature. Therefore, the expected effects^11^ of the finite experimental temperature (<20 nK; Methods) would be at most on the order of the QMC error bars.

**Fig. 3:**
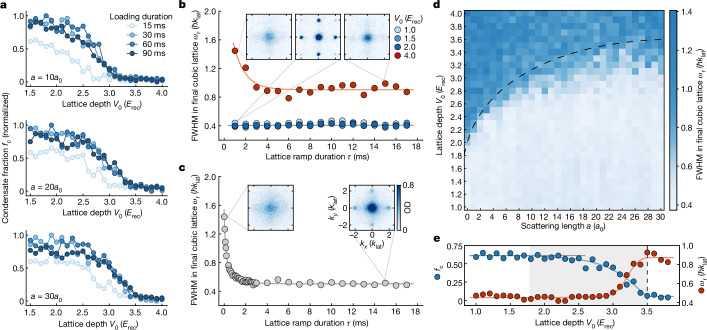
Non-adiabaticity of the Bose glass. **a**, Condensate fraction in the 2D quasicrystal (normalized within each plot) as a function of lattice depth for different loading durations and scattering lengths. Although 15-ms ramps result in consistently lower condensate fractions, there is no consistent difference between 30 ms and longer ramps. **b**, FWHM of the central peak (*w*_r_) after a linear ramp of duration *τ* from the 2D quasicrystal into a regular 3D cubic lattice, where the ground state is a superfluid. The coloured circles correspond to different depths of the quasicrystalline potential *V*_0_ for a fixed scattering length of *a* = 10*a*_0_. For ${V}_{0} < {V}_{{\rm{loc}}}^{(10)}$ (blue circles), the initial state in the quasicrystal is superfluid and the final states show strong superfluid order for all explored ramp times. For a deep Bose glass at ${V}_{0} > {V}_{{\rm{loc}}}^{(10)}$ (red circles), in contrast, there is no initial coherence and only a very limited degree of phase coherence can be restored, demonstrating the absence of adiabatic evolution into and out of the Bose glass. **c**, An equivalent measurement for a Mott insulator in a regular 3D cubic lattice (*V*_*x*,*y*,*z*_ = 16 *E*_rec_, *a* = 150*a*_0_). Although the initial state also lacks coherence, it can be rapidly restored by ramping down the lattice depth in *τ* ≳ 2 ms. Insets in **b** and **c** show TOF images and OD denotes the optical density. **d**, Phase diagram showing *w*_r_ for a slow ramp with *τ* = 15 ms highlighting three different regimes: a pure superfluid (light blue), an intermediate regime where superfluid and Bose glass coexist in the trap, and finally the pure Bose glass (darker blues). The transition into the pure Bose glass is consistent with the phase boundary extracted in Fig. 2b (dashed line). **e**, Comparing condensate fraction *f*_c_ in the quasicrystal with *w*_r_ for *a* = 23*a*_0_, demonstrating the consistency of all observations. The dashed line denotes the critical lattice depth ${V}_{{\rm{loc}}}^{(23)}$ extracted from the main diagram of Fig. 2b and the grey area indicates the intermediate regime where superfluid and Bose glass coexist. The solid lines in **b**, **c** and **e** are guides to the eye.

As a complementary observable that highlights the inhomogeneous nature of the system, the inset of Fig. 2b shows the same phase diagram analysed in terms of the condensate fraction ${f}_{{\rm{c}}}:= {{\mathcal{N}}}_{{\rm{coh}}}\,/{\mathcal{N}}$, that is, the number of atoms in the sharp interference peaks ${{\mathcal{N}}}_{{\rm{coh}}}$ divided by the total atom number ${\mathcal{N}}={{\mathcal{N}}}_{{\rm{coh}}}+{{\mathcal{N}}}_{{\rm{incoh}}}$, where ${{\mathcal{N}}}_{{\rm{incoh}}}$ represents the population of the incoherent background (see Methods for details). The condensate fraction is high for shallow lattices and begins to slowly decrease after the lattice depth exceeds the non-interacting critical depth ${V}_{{\rm{loc}}}^{(0)}$ (see also Fig. 3e). This initially small downwards trend gradually becomes stronger, and the condensate fraction eventually reaches zero at the same critical depth ${V}_{{\rm{loc}}}^{(a)}$ extracted from the FWHM measurement (dashed line).

The gradual decrease in the condensate fraction is consistent with the expected coexistence of superfluid and Bose glass in the system. This is the result of the inhomogeneous atomic density caused by the background harmonic dipole trap, as illustrated in Fig. 2c: when atoms are loaded into the lattice, the low-density edge of the cloud, where interaction effects vanish, will start to localize at the critical depth for non-interacting atoms ${V}_{{\rm{loc}}}^{(0)}$ (ref. ^37^). As we further increase the lattice depth, the phase boundary that separates the Bose glass from the superfluid core will slowly move towards higher densities until all atoms are ultimately in the Bose glass phase.

## Absence of adiabaticity at the Bose glass transition

In typical quantum phase transitions between ergodic phases, for example, from superfluid to Mott insulator^45^, an important experimental check is whether the phase transition was crossed adiabatically, and thereby reversibly, or whether the observed loss of coherence results from irreversible heating, due to, for instance, rapid non-adiabatic changes that generate entropy. In the present case, however, the situation is potentially rather different, as the Bose glass is expected to be non-ergodic such that the thermodynamic notion of adiabatic changes may not apply.

To investigate this, we first study in Fig. 3a the effect of different lattice loading durations on the resulting condensate fraction. A too-rapid lattice ramp (15 ms) gives rise to considerable heating already in the superfluid regime, leading to lower condensate fractions compared with slower ramps. Once the loading duration exceeds 30 ms, it however becomes irrelevant and the condensate fraction is independent of the loading rate, demonstrating adiabaticity within the superfluid and consistent critical lattice depths ${V}_{{\rm{loc}}}^{(a)}$.

To study whether the phase transition was crossed adiabatically, we next try to restore superfluid coherence. Here we first load the atoms into the 2D quasicrystalline lattice in 45 ms, and then continuously transform the non-periodic lattice into a periodic simple-cubic three-dimensional (3D) lattice. This transformation is carried out by linearly ramping the depth of the *x*, *y* and *z* lattices to 8*E*_rec_ over various durations *τ* while simultaneously reducing the depth of the remaining two diagonal lattices (Fig. 1a) to zero. The 3D cubic lattice was chosen as in this lattice the ground state is a superfluid with a finite critical temperature for condensation for all studied parameters^49^.

Figure 3b shows the FWHM of the central peak, *w*_r_, in the final periodic lattice for different ramp times *τ* at a fixed scattering length (*a* = 10*a*_0_), and the outcome highlights the fundamentally distinct behaviours of the superfluid and Bose glass phases. For ${V}_{0} < {V}_{{\rm{loc}}}^{(10)}$ (blue circles), the system remained superfluid during the entire sequence, and the ground state can adapt rapidly from a quasiperiodic extended wave to a periodic Bloch wave, as indicated by the sharp and narrow diffraction peaks for all ramp durations. For ${V}_{0} > {V}_{{\rm{loc}}}^{(10)}$ (red circles), however, where the system has entered the Bose glass regime, the initial state contains only very short-range coherence and hence results in a high *w*_r_. Furthermore, the measured *w*_r_ remains significantly above that of the superfluid even for the slowest ramps explored in this measurement. This demonstrates that the system in this regime can restore only a very limited degree of phase coherence and thereby directly highlights the significant entropy production arising from traversing, that is, entering, ramping through and exiting the Bose glass. In combination, the above measurements demonstrate that despite the loading duration becoming irrelevant for sufficiently slow ramps, it remains impossible to traverse the Bose glass isentropically, that is, in a thermodynamically adiabatic fashion.

To show that the reduced coherence is not solely caused by dynamical effects such as Kibble–Zurek-type dynamics^47^ during too-fast final ramps, Figure 3c shows an equivalent measurement starting from a Mott insulator in a deep 3D simple-cubic lattice, where phase coherence is recovered by reducing the lattice potential to the same final depth as in the previous case. In this case, sharp interference patterns can be recovered already within 2 ms of ramp-down time, consistent with previous observations^45,47^. This contrast not only experimentally confirms that the incoherent localized phase we observe in the optical quasicrystal is distinct from a Mott insulator but also highlights that the inability to traverse the Bose glass adiabatically is rather distinct from the critical slowing down expected at conventional continuous phase transitions^46,47^. It is consistent with glassy dynamics in general and the expected non-ergodic nature of the Bose glass in particular.

Figure 3d shows the FWHM of the central peak (*w*_r_) after a slow final ramp of *τ* = 15 ms and demonstrates that the observed breakdown of adiabaticity indeed coincides with the transition into the Bose glass. This is further corroborated by the cuts shown in Fig. 3e: as more and more atoms localize and enter the Bose glass, not only does the condensate fraction decrease but also the coherence cannot be restored.

## Conclusion

In this work, we experimentally study the 2D Bose glass in an optical quasicrystal with eight-fold rotational symmetry by probing the coherence properties of the system. We directly observe the phase transition between the Bose glass and the superfluid, in good agreement with QMC simulations^6^. In addition, we study the possibility to traverse the Bose glass adiabatically and always find significant entropy increases that are consistent with the expected non-ergodic character of the Bose glass. This paves the way for testing the connection between the Bose glass, MBL and glassy dynamics more generally. Quasicrystalline and quasiperiodic lattices offer a unique route to study MBL, as their long-range ordered nature can exclude conventional ergodic rare regions^41,50^ that are expected to destabilize MBL by seeding thermalization avalanches in real random systems^51,52^.

## Methods

### Experimental sequence

The experimental sequence begins with loading an almost pure Bose–Einstein condensate of about 1.2 × 10^5^ ^39^K atoms without discernible thermal fraction from a red-detuned crossed optical dipole trap (*λ*_dip_ = 1,064 nm, with trap frequencies (*ω*_*x*_, *ω*_*y*_, *ω*_*z*_) = 2π × (55, 43, 330) Hz) into a blue-detuned 2D quasiperiodic optical lattice (*λ*_lat_ = 725.4 nm). The initial temperature is bounded from above by *T*_i_ < 20 nK due to a conservative lower bound on the observed condensate fraction. Even neglecting that temperatures for weakly interacting bosons typically decrease when loading into a lattice, the resulting change in critical chemical potential Δ*μ* due to the finite temperature is small according to ref. ^11^, that is, Δ*μ*/*μ* < 2.5% or, equivalently, Δ*μ*/(*μ* − *ϵ*_0_) < 20%, where ${{\epsilon }}_{0}:= \min \,{{\epsilon }}_{i}$. During the loading, the individual lattice depths are increased in 45 ms from 0 to their target values using exponential ramps with a time constant of 10 ms. The used target depths for the four horizontal lattices range within *V*_0_ = 1–4*E*_rec_ while a fixed depth of *V*_*z*_ = 20*E*_rec_ for the vertical *z* lattice ensures the formation of well-defined quasi-2D systems. After this ramp, the atoms are held in the quasicrystal for 10 ms. For imaging, we apply a short ‘booster stage’^53^ before we switch off all trapping potentials and record the matter-wave interference pattern by taking an absorption image after 9 ms TOF.

**Extended Data Fig. 1:**
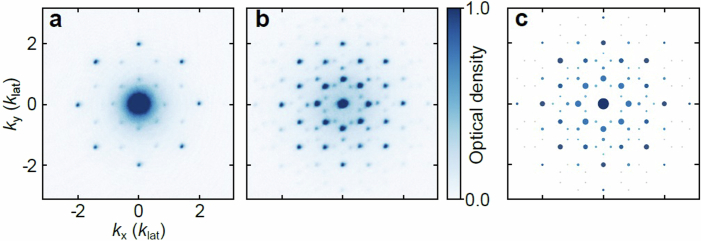
Effect of booster stage (*V*_0_ = 2 *E*_rec_, *a* = 10 *a*_0_) and an example of the population extraction. **a**, In the absence of the booster, the majority of condensed atoms remain in the central diffraction peak, with only a small fraction occupying the satellite peaks. The high atomic density of the central peak causes almost all the imaging light around this central area to be fully absorbed, leading to significant imaging saturation at **k** = 0. **b**, the booster stage promotes condensed atoms to higher diffraction orders, thus facilitating the fitting. **c**, Simulated diffraction pattern for the first 6 diffraction orders. The 81 peaks considered in the population count are coloured in blue, with their radius indicating the extracted population *n*_*k*_. Gray dots represent the peaks that can also be observed but are not included in the population count due to their low populations. Images in (a,b) are averaged over 30 experimental shots in order to visually emphasise the signal from very weakly populated high-order diffraction peaks.

The booster stage consists of linearly increasing the potential depth of the horizontal lattices in 40 μs to a final depth of *V*_final_ = 6*E*_rec_. This stage is sufficiently short to not change the coherence properties of the system while providing a tighter on-site confinement and thereby not only enhancing the brightness of high-order diffraction peaks but also significantly reducing the heavy saturation on the central momentum peak (Extended Data Fig. 1a,b).

The interaction strengths *U*_*i*_ ∝ *a* are independently controlled by tuning the atomic s-wave scattering length (*a*) using the Feshbach resonance close to 403 G of the $| F=1,{m}_{F}=1\rangle$ state in ^39^K (refs. ^54,55^). Here *F* denotes the total angular momentum and *m*_*F*_ denotes the magnetic quantum number of the state. To ensure broadly comparable density distributions, the scattering length is initially prepared at a common finite value of *a* = 12*a*_0_ before the lattice loading starts and is then changed using a 20 ms linear current ramp to the desired value within *a* = 0–30*a*_0_ starting after the first 5 ms of the lattice ramp. Subsequently, the scattering length remains constant until being suddenly switched to *a* = 0*a*_0_ at the beginning of the TOF.

A periodic cubic 3D lattice can be produced by using only two orthogonal 1D lattices (*x*, *y*) out of the four in-plane 1D lattices indicated in Fig. 1 as well as the perpendicular *z* lattice. This was used as the final lattice in the attempt to restore superfluid coherence. For the final lattice depths in Fig. 3b–e, the ground state in the cubic lattice is a superfluid for all studied interactions. Although the cubic lattice is a priori only one of several possible choices, ramping into a periodic 3D lattice has the advantage that it results in an ergodic system where long-range coherence emerges below a finite critical temperature^49^.

Furthermore, the same cubic lattice geometry was also used for preparing the initial Mott insulating state in Fig. 3c, where the restoration of phase coherence is then carried out by employing a 16–8*E*_rec_ linear ramp on all the three lattice axes simultaneously.

### Coherence length and extraction of condensate fraction

In the TOF images, the width of the sharp diffraction peaks of the superfluid is dominated by the finite initial cloud size, which in combination with the finite TOF acts as an effective resolution limit for the measured momentum distribution^47^. Therefore, no significant broadening is expected as long as the coherence lengths in the superfluid part remains above 3– 5*λ*_lat_ (ref. ^47^). In the inhomogeneous system, the FWHM of the central peak (compare Fig. 2b) corresponds to this resolution-limited width as long as the *k* ≈ 0 peak of the superfluid remains visible atop the incoherent background of localized atoms. The FWHM jumps to the background width once the interference peaks have completely merged into the background, thereby giving rise to the sharp signature shown in Fig. 2b. This jump hence stems from the combination of the inhomogeneous system with the effective resolution limit imposed by the finite TOF and would not be present in a homogeneous system.

The condensate fraction *f*_c_ is a complimentary observable that measures the fraction of coherent atoms and is evaluated for every shot according to ${f}_{{\rm{c}}}={{\mathcal{N}}}_{{\rm{coh}}}\,/{\mathcal{N}}$, where ${{\mathcal{N}}}_{{\rm{coh}}}$ is the population in the sharp interference peaks, and ${\mathcal{N}}={{\mathcal{N}}}_{{\rm{coh}}}+{{\mathcal{N}}}_{{\rm{incoh}}}$ is the total atom number with ${{\mathcal{N}}}_{{\rm{incoh}}}$ being the number of atoms in the incoherent background.

To extract ${{\mathcal{N}}}_{{\rm{coh}}}={\sum }_{k}{n}_{k}$ from the TOF images, we first identify the most pronounced 81 diffraction peaks within the first six diffraction orders^5^ and then extract their populations *n*_*k*_ by fitting independent 2D Gaussian profiles to each peak. To prevent counting spurious populations from weakly populated peaks, we exclude fitted populations *n*_*k*_ below 0.12% of the total atom number. Extended Data Fig. 1c illustrates the extracted populations.

The atom number in the incoherent background, ${{\mathcal{N}}}_{{\rm{incoh}}}$, is acquired by performing an additional 2D Gaussian fit to the whole cloud (region of interest $3.3\times 3.3\,{(\hbar {k}_{{\rm{lat}}})}^{2}$), where all detected diffraction peaks were masked during the fitting.

### Parameter calibration

The two main experimental parameters are the lattice depth and the scattering length between atoms. The lattice depth is calibrated to within 0.1*E*_rec_ by analysing the dynamics of Kapitza–Dirac diffraction for each 1D lattice individually; see the supplementary material of ref. ^5^ for details.

The scattering length is calibrated by observing the prominent atom-loss features corresponding to the zero-crossing of the scattering length, where the in situ density is highest, and the Feshbach resonance, where the loss coefficient is maximal. We then interpolate the scattering length between them using the common functional form^55,56^. As an independent cross-check, the magnetic field is calibrated using radio-frequency spectroscopy of the $| F=1,{m}_{F}=-1\rangle$ to $| F=1,{m}_{F}=0\rangle$ transition in ^87^Rb and converted to a scattering length using literature values for the parameters of the Feshbach resonance^55,56^. The two approaches agree to ≲1*a*_0_.

### Comparing with QMC simulations

**Extended Data Fig. 2:**
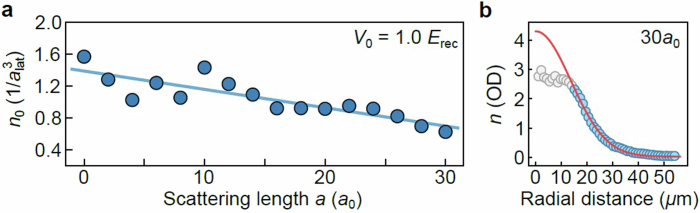
Extraction of central density. **a**, Central density as a function of scattering length. **b**, Fit to in-situ column density distribution used to extract the effective cloud width for calculating the central density. The employed absorption imaging starts to saturate for OD ≳ 2.5, hence, data points above this value (grey) have been excluded from the fit.

**Extended Data Fig. 3:**
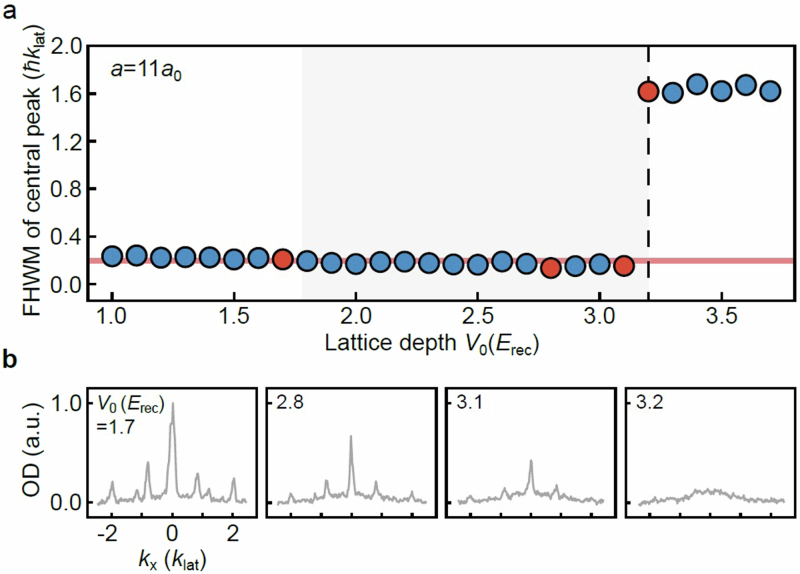
Width of central peak. **a**, FWHM of central peak for *a* = 11*a*_0_, data taken from Fig. 2b. **b**, Normalised cuts through the density distribution observed after time-of-flight (analogous to cuts through Fig. 2a). The red line in **a** indicates the measured in-situ cloud size, demonstrating that the observed peak width is dominated by the in-situ size. Both the FWHM and the absence of thermal components around the peaks in **b** demonstrate that the momentum distribution is compatible with *T* = 0 up to very close to the phase transition. Red dots in upper panel indicate the lattice depths used in the lower panel.

The QMC calculations reported in ref. ^6^ were performed as a function of the density *n* in a homogeneous system at fixed interaction strength *g*. As the main panel of Fig. 2b focuses on the phase transition in the centre of the trap, we extract the experimental central density *n*_0_ from in situ absorption images using the known aspect ratio of the trap. To minimize statistical noise, we measure *n*_0_ at different scattering lengths (*a* = 0–30*a*_0_) and constant lattice depth (*V*_0_ = 1*E*_rec_) and find a mild interaction dependence *n*_0_(30*a*_0_) ≈ 1/2 *n*_0_(0*a*_0_) for the used lattice ramp (Extended Data Fig. 2). In addition, we relate the 2D interaction coupling constant *g* used in ref. ^6^ back to the 3D scattering length *a* via

$$\begin{array}{c}g=\frac{{{\hslash }}^{2}}{m}\mathop{g}\limits^{ \sim }\,,\,\mathop{g}\limits^{ \sim }\approx {\mathop{g}\limits^{ \sim }}_{0}=\frac{2{\rm{\pi }}}{{\rm{l}}{\rm{n}}({a}_{{\rm{l}}{\rm{a}}{\rm{t}}}/{a}_{2{\rm{D}}})},\\ {a}_{2{\rm{D}}}=2.092\,{l}_{\perp }\exp \left(-\sqrt{\frac{{\rm{\pi }}}{2}}\frac{{l}_{\perp }}{a}\right).\end{array}$$

Here *a*_lat_ = *λ*_lat_/2 and ${l}_{\perp }=\sqrt{\hbar /m{\omega }_{\perp }}$ is the characteristic confining length given by the strong *z* lattice with a trapping frequency of *ω*_⊥_ = 2π × 87 kHz.

### Excluding Mott insulators

To investigate the possibility of Mott insulators in our experiment, we numerically compute the Bose–Hubbard parameters of the quasiperiodic potential using the results from ref. ^43^. We calculate the site-dependent ratio between on-site interactions and tunnelling energies ${U}_{i}/{\sum }_{j}| \,{J}_{ij}|$, where the sum runs over all significant tunnelling elements linking site *i* to other adjacent sites. Within the explored parameter regime, this ratio reaches a maximum of $\max ({U}_{i}/{\sum }_{j}| \,{J}_{ij}| )\approx 1.4$ for the case of *a* = 30*a*_0_ and *V*_0_ = 4.0*E*_rec_. This is significantly below the critical interaction strength for forming a Mott insulator in a 2D square lattice (*U*/*zJ*)_c_ ≈ 4.385 (ref. ^57^), where *z* = 4 represents the number of nearest neighbours. Furthermore, the studied parameter range lies within the weakly interacting regime of ref. ^11^, and Mott insulators can hence be excluded in this experiment.

## Online content

Any methods, additional references, Nature Portfolio reporting summaries, source data, extended data, supplementary information, acknowledgements, peer review information; details of author contributions and competing interests; and statements of data and code availability are available at 10.1038/s41586-024-07875-2.

## Data Availability

The data shown in this paper are available from 10.17863/CAM.111477.
